# *LEO1* haploinsufficiency is associated with developmental delays and autism spectrum disorder

**DOI:** 10.1038/s10038-025-01410-5

**Published:** 2025-09-24

**Authors:** Emilie C. Ung, Nicholas A. Borja

**Affiliations:** 1 John P. Hussman Institute for Human Genomics, University of Miami Miller School of Medicine, Miami, FL USA; 2 John T. Macdonald Foundation Department of Human Genetics, University of Miami Miller School of Medicine, Miami, FL USA

**Keywords:** Autism spectrum disorders, Genetics research

## Abstract

*LEO1* encodes a core subunit of the evolutionarily conserved RNA polymerase associated factor 1 complex (PAF1C), a key regulator of eukaryotic gene expression. While burden analyses suggest an association between rare *LEO1* variants and an increased risk for neurodevelopmental disorder, the paucity of reported cases has prevented a definitive characterization of the resulting phenotype. We describe a male child with a novel de novo frameshift variant in *LEO1* c.446dup (p.Asp149Gluf s*2) and undertake a comprehensive phenotype delineation of all previously reported patients. Developmental delay and autism spectrum disorder were core features common across patients with truncating variants, though rarer manifestations were also observed. This analysis supports *LEO1* haploinsufficiency as a mechanism for this neurodevelopmental disorder. Further research is needed to more completely ascertain its associated features and penetrance. We nevertheless encourage its recognition as a definitive disease gene and inclusion in multigene panels.

*LEO1* encodes a core subunit of the evolutionarily conserved RNA polymerase associated factor 1 complex (PAF1C), a key regulator of eukaryotic gene expression [1]. PAF1C physically associates with RNA polymerase II and is integral to the transcription cycle, including elongation, mRNA 3’ end formation, processing, and chromatin modification [2]. The complex also orchestrates RNA polymerase II degradation in response to DNA damage [3]. Despite their operating as a complex, each PAF1C subunit has functional specialization, with LEO1 regulating heterochromatin stability and histone H3 turnover during cellular quiescence [4]. In addition, LEO1 is critical during embryogenesis in model organisms, where homozygous knockout of *leo1* in zebrafish leads to severe defects in cardiac and neural crest development and in mice is associated with preweaning lethality [5, 6].

Burden analyses in cohorts of neurodevelopmental disorder patients have identified significant enrichment for rare variants in *LEO1* [7–9]. This association is supported by the substantial fraction of neurodevelopmental disorders in humans occurring in genes within transcription and chromatin remodeling pathways, including other PAF1C subunits [8, 10, 11]. Nevertheless, the reported *LEO1* variants to date have included a combination of truncating variants expected to result in a loss-of-function, missense variants, and paternally inherited promoter deletions that paradoxically appear to cause gene overexpression [9, 12, 13]. In addition to the paucity of reported cases, the limited clinical data available for each proband has prevented a more definitive characterization of the resulting phenotype.

Here, we report the case of a male child with a de novo frameshift variant in *LEO1*, providing further evidence that haploinsufficiency of this gene is associated with an increased risk for neurodevelopmental impairment. This is reinforced by the comprehensive phenotype delineation we undertake of all previously reported patients.

The proband is the first liveborn child to a nonconsanguineous couple of Cuban and Japanese descent. He was born at 30 weeks gestation following a pregnancy complicated by intrauterine growth restriction, with a weight of 1.0 kg (*z* = −1.24), length of 38 cm (*z* = -0.48), and head circumference of 28 cm (*z* = 0.32). During his admission to the NICU he developed respiratory distress and apnea of prematurity, which was managed with oxygen supplementation. Transthoracic echocardiogram revealed normal biventricular size and systolic function. He was discharged home at 2 months of age. At 18 months old he was still not using any words. The brain MRI performed at that time was interpreted as normal. He also had persistent spit-ups with feeding, though evaluation with a swallow study and esophagram found no impairment, indicating a functional disorder. He was ultimately diagnosed with autism spectrum disorder, as he exhibited oral aversion to foods, deficits in social communication, and fine motor impairments, for which he received feeding, speech, and occupational therapies, respectively. By age 5 he was able to feed himself independently, speak in complete sentences, read multi-syllabic words, count to ten, identify colors, and trace letters. His medical history was otherwise only significant for reactive airway disease and recurrent respiratory infections requiring multiple hospitalizations. Physical examination did not reveal craniofacial dysmorphisms or physical anomalies. Neither his parents nor his seven half-siblings are similarly affected.

His genetic workup included chromosomal microarray and Fragile X testing, which was unrevealing. Trio exome sequencing of the proband and his unaffected parents was performed at an accredited commercial laboratory, GeneDx (Gaithersburg, MD). This identified that he is heterozygous for a single base pair duplication in exon 2 of *LEO1* (NM_138792.4:c.446dup) (NP_620147.1:p.Asp149Gluf s*2). No other variants were reported. Our analysis found the *LEO1* variant is absent in the Genome Aggregation Database (gnomAD v4.1.0) (PM2) [14]. Neither of his confirmed parents harbored the variant, establishing that it occurred de novo in our proband (PS2). As such, we determined that our proband’s *LEO1* variant meets criteria for a likely pathogenic classification using the ACMG guidelines for the interpretation of variants [15].

We next compared the clinical history of our proband to the six previously reported patients with *LEO1* truncating variants (Table 1). Analysis of the variant position within the gene suggested that each would lead to nonsense-mediated decay of the mRNA (Supplementary Fig. 1). Three of these previously reported truncating variants were also confirmed to have occurred de novo, providing additional evidence of their pathogenicity. A consistent phenotype in our proband and the previously described patients was observed, with neurodevelopmental impairments that included developmental delay, autism spectrum disorder, and intellectual disability in the absence of structural brain anomalies. Congenital hypotonia as well as ADHD were also described in a minority of cases, and one patient had a history of microcephaly, seizures and an abnormal gait [16]. Additional manifestations described in only single cases included extraocular muscle dysfunction, disordered sleep, cryptorchidism, and proximal muscle weakness.

**Table 1:** Comparison of our proband’s phenotype with all previously reported affected individuals heterozygous for a *LEO1* truncating variant as well as non-truncating variant

LEO1-related features	Proband	Reported truncating variants (*n* = 6)	Reported non-truncating variants (*n* = 9)
MOLECULAR			
DNA and protein change NM_138792.4 (NP_620147.1)	c.446dup (p.Asp149Glufs*2)	c.1663 C > T (p.Arg555*) c.1140del (p.Leu382Serfs*3) c.1276 C > T (p.Arg426*) c.187 G > T (p.Gly63*) c.1363 G > T (p.Glu455*) c.814+1 G > A (intronic)	c.1109 G > A (p.Gly370Glu) c.1478 C > G (p.Pro493Arg) c.1411 C > A (p.Leu417Ile) c.122 C > T (p.Ser41Phe) c. 1519 T > C (p.Ser507Pro) c.1148 G > T (p.Ser383Ile) *LEO1* promoter deletion in 3 cases
Inheritance	De novo	De novo (50%), Paternal (17%) Unknown (33%)	De novo (67%), Paternal (33%)
Sex	Male	Male (67%), Female (16.5%), Unknown (16.5%)	Male (56%), Female (44%)
NEURODEVELOPMENTAL			
Autism spectrum disorder	+	4 (67%)	8 (89%)
Developmental delay	+	4 (67%)	4 (44%)
Hypotonia	−	2 (33%)	1 (11%)
Intellectual disability	−	3 (50%)	1 (11%)
ADHD	−	1 (16.5%)	1 (11%)
Feeding impairment	+	0 (0%)	0 (0%)
Abnormal gait	−	0 (0%)	2 (22%)
Seizures	−	1 (16.5%)	0 (0%)
Abnormal brain MRI	−	0 (0%)	2 (22%)
GROWTH/SKELETAL			
Short stature	−	0 (0%)	0 (0%)
Failure to thrive	−	0 (0%)	0 (0%)
Microcephaly	−	1 (16.5%)	0 (0%)
Craniosynostosis	−	0 (0%)	1 (11%)
Craniofacial dysmorphisms	−	1 (16.5%)	1 (11%)
Joint laxity	−	1 (16.5%)	1 (11%)
Pes planus	−	0 (0%)	2 (22%)
MISCELLANEOUS FEATURES			
Extraocular muscle dysfunction	−	1 (16.5%)	1 (11%)
Hearing impairment	−	0 (0%)	1 (11%)
Cardiac anomalies	−	0 (0%)	1 (11%)
Disordered sleep	−	1 (16.5%)	2 (22%)
Recurrent infections	+	0 (0%)	1 (11%)
Constipation	−	0 (0%)	1 (11%)
Cryptorchidism	−	1 (16.5%)	0 (0%)
Proximal muscle weakness	−	1 (16.5%)	0 (0%)

Finally, our analysis was expanded to include the nine previously reported patients with missense and splice site variants, as well as promoter deletions (Table 1). In three of these cases, the *LEO1* variant was inherited from an unaffected parent, with the remainder occurring de novo. These patients had a broader range of clinical findings that included craniosynostosis and craniofacial dysmorphisms, as well as cardiac anomalies and hearing loss, albeit in a minority of cases. Their most frequent manifestations, however, also included developmental delay, autism spectrum disorder, and intellectual disability, overlapping with the truncating variant patients.

Based on this detailed clinical characterization of a proband and independently ascertained cases with de novo loss-of-function variants, we conclude that *LEO1* haploinsufficiency is responsible for a neurodevelopmental disorder. This is further supported by the gene’s intolerance to loss-of-function variants in gnomAD v4.1.0 (pLI = 0.99, LOEUF = 0.49). In addition, we provide the initial delineation of the *LEO1*-related phenotypic spectrum, which includes core features common across variant types, as well as less common manifestations that may be variant-specific or, conversely, unrelated to the disorder. Larger cohorts of affected patients will need to be described to better define the broader neurodevelopmental risks of the *LEO1*-related syndrome and its associated penetrance, as the lack of clinical data in previous reports represents a significant limitation to current estimates. Functional studies should work towards describing how *LEO1* haploinsufficiency leads to transcriptional dysregulation and more clearly demonstrate the molecular consequences of non-truncating variants, including promoter deletions. Nevertheless, we recommend that *LEO1* be recognized as a disease gene with strong evidence of causation and be included in multigene panels for developmental delay and autism spectrum disorder.

## Supplementary information

Supplementary Figure 1
