# Supplementary material for: LEO1 haploinsufficiency is associated with developmental delays and autism spectrum disorder
Source: J Hum Genet. 2025 Sep 24;71(2):109–11. doi: 10.1038/s10038-025-01410-5 (PMC12765696; doi:10.1038/s10038-025-01410-5)
Supplement: Supplementary file 1 — Supplementary Figure 1 [file 10038_2025_1410_MOESM1_ESM.docx]

**Supplementary Figure 1. Lollipop plot of proband’s variant and all previously reported truncating variants** mapped to LEO1 transcript NM_138792.4 with exons separated by spaces. Each variant is labeled with the name of the first author of the publication where it was initially reported. All variants below are expected to trigger nonsense mediated decay as they are located upstream of the final exon and 50 nucleotides upstream of the penultimate exon.^1^ Figure generated using ProteinPaint, an application available through St. Jude Cloud.^2^

**
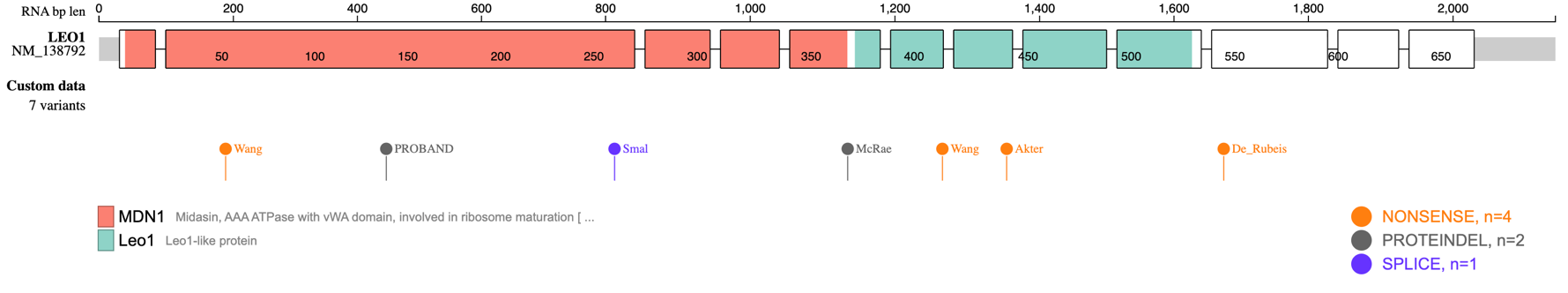
**

**Supplementary References**

1. Nagy E, Maquat LE. A rule for termination-codon position within intron-containing genes: when nonsense affects RNA abundance. Trends Biochem Sci. 1998;23:198-9.

2. Zhou X, Edmonson MN, Wilkinson MR, Patel A, Wu G, Liu Y, et al. Exploring genomic alteration in pediatric cancer using ProteinPaint. Nat Genet. 2016;48:4-6.
